# One-year outcomes in sepsis: a prospective multicenter cohort study in Japan

**DOI:** 10.1186/s40560-025-00792-0

**Published:** 2025-05-01

**Authors:** Keibun Liu, Shinichi Watanabe, Kensuke Nakamura, Hidehiko Nakano, Maiko Motoki, Hiroshi Kamijo, Matsuoka Ayaka, Kenzo Ishii, Yasunari Morita, Takashi Hongo, Nobutake Shimojo, Yukiko Tanaka, Manabu Hanazawa, Tomohiro Hamagami, Kenji Oike, Daisuke Kasugai, Yutaka Sakuda, Yuhei Irie, Masakazu Nitta, Kazuki Akieda, Daigo Shimakura, Hajime Katsukawa, Toru Kotani, David McWilliams, Peter Nydahl, Stefan J. Schaller, Takayuki Ogura, the ILOSS Study Group

**Affiliations:** 1 Non-Profit Organization ICU Collaboration Network (ICON), Tokyo, Japan; 2 Department of Physical Therapy, Gifu University of Health Science, Gifu, Japan; 3 Department of Rehabilitation, National Hospital Organization Nagoya Medical Center, Nagoya, Aichi Japan; 4 Department of Critical Care Medicine, Yokohama City University Hospital, 3-9, Fukuura, Kanazawa-Ku, Yokohama, Kanagawa 236-0004 Japan; 5 Department of Emergency and Critical Care Medicine, Hitachi General Hospital, Hitachi, Ibaraki Japan; 6 Department of Emergency and Critical Care Medicine, Shinshu University School of Medicine, Nagano, Japan; 7 Department of Emergency and Critical Care Medicine Faculty, Saga University Hospital, Saga, Saga Japan; 8 Department of Anesthesiology, Intensive Care Unit, Fukuyama City Hospital, Fukuyama, Hiroshima Japan; 9 Department of Emergency and Intensive Care Medicine, National Hospital Organization Nagoya Medical Center, Nagoya, Aichi Japan; 10 Department of Emergency, Critical Care, and Disaster Medicine, Okayama University Graduate School of Medicine, Dentistry, and Pharmaceutical Sciences, 2-5-1 Shikata-Cho, Okayama Kita-Ku, Okayama, 700-8558 Japan; 11 Department of Emergency, Okayama Saiseikai General Hospital, 2-25 Kokutaityo, Okayama Kita-Ku, Okayama, 700-8511 Japan; 12 Emergency and Critical Care Medicine, Faculty of Medicine, University of Tsukuba, Ibaraki, Japan; 13 Department of Emergency, Tsukuba Medical Center Hospital, Tsukuba, Ibaraki Japan; 14 Department of Rehabilitation, Japan Red Cross Narita Hospital, Narita, Chiba Japan; 15 Tajima Emergency & Critical Care Medical Center, Toyooka Public Hospital, Toyooka, Hyogo Japan; 16 Department of Rehabilitation, Tsuchiura Kyodo General Hospital, Tsuchiura, Ibaraki Japan; 17 Department of Emergency and Critical Care Medicine, Nagoya University Graduate School of Medicine, Nagoya, Aichi Japan; 18 Department of Intensive Care Medicine, Okinawa Kyodo Hospital, Naha, Okinawa Japan; 19 Department of Emergency and Critical Care Medicine, Fukuoka University Hospital, Fukuoka, Fukuoka Japan; 20 Department of Intensive Care Unit, Niigata University Medical and Dental Hospital, Niigata, Niigata, Japan; 21 Department of Emergency Medicine, SUBARU Health Insurance Society Ota Memorial Hospital, Ota, Gunma Japan; 22 Graduate School of Data Science, Shiga University, Shiga, Japan; 23 Japanese Society for Early Mobilization, Tokyo, Japan; 24 Showa Medical University, Shinagawa, Tokyo Japan; 25 Centre for Care Excellence, Coventry University, Coventry, UK; 26 Critical Care, University Hospitals Coventry & Warwickshire NHS Trust, Coventry, UK; 27 Nursing Research, University Hospital Schleswig-Holstein, Kiel, Germany; 28 Institute of Nursing Science and Development, Paracelsus Medical University, Salzburg, Austria; 29 Department of Anesthesia, Intensive Care Medicine and Pain Medicine, Clinical Division of General Anesthesia and Intensive Care Medicine, Medical University of Vienna, Wien, Austria; 30 Department of Anesthesiology and Intensive Care Medicine (CCM/CVK), Charité - Universitätsmedizin, Berlin, Germany; 31 Department of Emergency Medicine and Critical Care Medicine, Tochigi Prefectural Emergency and Critical Care Center, Saiseikai Utsunomiya Hospital, Utsunomiya, Tochigi Japan; 32 Present Address: 2-15-13 Hongo, Bunkyo-Ku, Tokyo, 113-0033 Japan

**Keywords:** Intensive care unit, Morbidity, Mortality, Post-intensive care syndrome, Quality of life, Sepsis

## Abstract

**Background:**

Sepsis is a leading cause of death in intensive care units (ICU). Sepsis survivors are often left with significant morbidity, termed post-intensive care syndrome (PICS), impacting post-sepsis life. The aim was to present detailed data on the prognostic and functional long-term outcomes of ICU patients with sepsis in Japan, which is currently lacking and therefore prevents development of targeted solutions.

**Methods:**

A multicenter prospective study, involving 21 ICUs in 20 tertiary hospitals in Japan, included all consecutive adult ICU patients between November 2020 and April 2022, and diagnosed with sepsis at ICU admission (Sepsis 3). Follow-ups were performed at 3, 6, and 12 months after hospital discharge by telephone and mail. Primary outcome was death or incidence of PICS, defined by any of physical dysfunction (Barthel Index ≤ 90), cognitive dysfunction (Short Memory Questionnaire < 40), or mental disorder (any subscales for anxiety or depression of Hospital Anxiety and Depression Scale ≥ 8, or Impact of Event Scale-Revised ≥ 25). Secondary outcomes included Quality of Life (QOL), employment, and use of hospital, emergency, rehabilitation, and psychiatric services. A multivariable analysis investigated independent factors associated with each dysfunction at each follow-up.

**Results:**

A total of 339 patients were included (median age 74 [67–82] years, 60% male, 77% septic shock, and a median SOFA of 9 [6–12]). Mortality was 23% at hospital discharge, increasing to 37% at 12 months. The rate of death or those who met PICS Criteria at hospital discharge was 89%, with a death or PICS incidence of 73%, 64%, and 65% at 3, 6, and 12 months, respectively. Limited improvements in QOL and return to work (44%), high rates of hospital readmissions (40%), frequent emergency service usage (31%), and low utilization of rehabilitation and psychiatric services (15% and 7%) were identified over the first year. The incidence of any PICS-related dysfunction was consistently an independent factor for the incidence of the same dysfunction at the following follow-ups.

**Conclusions:**

This multicenter study identified the distinct realities of post-sepsis life in Japanese ICU patients, highlighting the unique challenges in improving their functions and returning to daily life.

*Trial Registration*

University Hospital Medical Information Network UMIN000041433

**Supplementary Information:**

The online version contains supplementary material available at 10.1186/s40560-025-00792-0.

## Background

Sepsis is a life-threatening condition characterized by an excessive immune response and subsequent organ dysfunction, resulting in high mortality and morbidity [1, 2]. Since 2020, a total of 48.9 million people worldwide have been affected, leading to 11 million sepsis-related deaths, which account for 20% of all global fatalities [3]. Due to advancements in sepsis management, survival rates have improved over the past few decades [4]. However, the morbidity experienced by sepsis survivors has been increasingly recognized, the impact of which extends beyond hospitalization and persists even after discharge, severely affecting essential life functions [5]. This long-term perturbation is known as Post-Intensive Care Syndrome (PICS), which is characterized by physical and cognitive impairments as well as mental disorders that can last for years following recovery from the Intensive Care Unit (ICU) [6–8]. PICS imposes a significant burden on the lives of patients post-sepsis, hindering their ability to return to their previous lives and jobs because of impaired activities of daily living (ADL) and a diminished quality of life (QOL) [9, 10] Given the increasing number of sepsis patients, along with the decreasing trend of mortality in sepsis [11], effective interventions to prevent or treat sepsis-related morbidity are urgently required.

The number of studies exploring post-sepsis recovery following ICU and hospital discharge has significantly increased, primarily from Western countries outside of Japan. The characteristics and backgrounds of ICU admissions vary widely by region. For instance, the ICU population reported in Western contexts tends to have a relatively high BMI and younger age compared to the typically older age and lower BMI of patients in Japan’s ICUs [12–14]. This heterogeneity in patient profiles may influence overall outcomes, posing challenges in interpreting studies from outside Japan that may not fully apply to the ICU population there. For example, multiple international studies have indicated that older ICU patients suffering from sepsis experienced significantly higher rates of mortality and morbidity, along with more severe functional impairment post-hospital discharge [15–17]. The Japanese Clinical Practice Guidelines for Management of Sepsis and Septic Shock 2020 (J-SSCG 2020) strongly emphasizes the importance of prevention and interventions to address PICS [18]. However, the evidence supporting these recommendations is derived from international studies, which involve different patient characteristics than those found in Japan, and therefore may not be applicable to the specific patient characteristics of critical care populations in Japan.

Consequently, we conducted a multicenter prospective cohort study involving 21 ICUs in tertiary hospitals across Japan, aiming to investigate sepsis-related outcomes, including mortality and the incidence of PICS, for up to one year after hospital discharge.

## Methods

### Study settings

This was a multicenter prospective cohort study conducted across 21 ICUs in 20 tertiary hospitals in Japan. Ethical approvals were obtained from all hospitals (central ethics committee: Saiseikai Utsunomiya Hospital, Approval number: 2019–72). Informed consents were obtained from all patients. This study was registered in UMIN (UMIN000041433) and adhered to the STROBE guideline. The protocol for this study was published previously [19].

### Patients

All consecutive patients admitted to the ICUs between 1 November 2020 and 30 April 2022 and who were diagnosed with sepsis or septic shock (according to the Sepsis-3 definition) at ICU admission were included. Exclusion criteria included patients who were under 18 years old, diagnosed with COVID-19, expected to be discharged from the ICU within 48 h, had a central nervous system disorder that was considered unrelated to sepsis based on clinical examination such as stroke, severe head trauma, brain tumor, hypoxic encephalopathy, cerebrovascular dementia, and Alzheimer’s disease, could not communicate due to pre-existing psychiatric symptoms, could not walk independently even with a walking aid prior to hospitalization, or were in end-of-life or terminal state at ICU admission which could contribute to the treatment limitations in and after ICU stay. Patients for whom consent forms could not be obtained were also excluded. All patients at the participating ICUs received standard management based on the national sepsis guideline, (i.e., sepsis management, sedation, analgesia, delirium, etc.). [18, 20, 21].

### Variables

The following data on patient characteristics were obtained at ICU admission, during the ICU stay, and at hospital discharge: age, sex, body mass index (BMI), Charlson Comorbidity Index (CCI)[22], Clinical Frailty Score (CFS) prior to hospital admission [23], Barthel Index [24] prior to hospital admission, recorded as the best value within the two weeks based on information from family members, employment status prior to hospital admission, ICU admission route, source of infection, Sequential Organ Failure Assessment (SOFA) sum score [25], presence of septic shock at ICU admission, lactate level at ICU admission, and the use of organ support during the ICU stay, including noninvasive positive pressure ventilation (NPPV), high-flow nasal cannula (HFNC), invasive mechanical ventilation (IMV), and renal replacement therapy (RRT), ICU mortality, duration of IMV, and the length of ICU and hospital stay. Those who met the PICS Criteria, as described in the following section of *Outcome measures,* were also evaluated at the time of hospital discharge.

### Outcome measures

Outcomes were assessed and obtained physically at hospital discharge and via telephone and mail at the 3-, 6-, and 12-month follow-ups after the hospital discharge by researchers from each hospital.

The primary outcome was a composite outcome as the rate of death (mortality) and the incidence of PICS, in consideration of the survival bias [26]. The incidence of PICS was defined by the presence of any of the following: (1) physical dysfunction, indicated by a score of 90 or lower on the Barthel Index (BI), (2) cognitive dysfunction, defined as a score of less than 40 on the Short Memory Questionnaire (SMQ) [27–29], or (3) a mental disorder, characterized by a score of 8 or higher on the anxiety or depression subscale of the Hospital Anxiety and Depression Scale (HADS) [30], or a score of 25 or higher on the Impact of Event Scale-Revised (IES-R) [31] at the follow-ups.

Among patients who survived to hospital discharge, (defined as survivors), the following secondary outcomes were obtained: the incidence of PICS, the incidence of each dysfunction, the score of each assessment, the number of patients with two of three or all three domains of PICS, EuroQoL 5-dimension 5-level (EQ-5D-5L) and visual analog scale (EQ-VAS) for the assessment of QOL [32], employment status, employment rates among those who were employed prior to hospitalization, body weight and changes compared to the baseline at ICU admission, readmissions to the hospital or ICU after discharge, unplanned emergency room visits, and the use of physical rehabilitation or psychiatric consultation clinics among survivors, as well as among those with physical dysfunctions or mental disorders, respectively, at each follow-up time point. The EQ-5D-5L was initially recorded as a 5-digit number and subsequently converted into the index value [27, 32, 33].

### Statistical methods

Continuous variables were described using the median and interquartile range (IQR) and compared with the Mann–Whitney *U* test. Categorical variables were expressed as numbers and percentages and compared using the Fisher exact test or the Chi-square test as appropriate. A multivariable logistic regression analysis was conducted to investigate the risk factors associated with each dysfunction at each follow-up time point. The covariates were selected as follows based on the clinically significant characteristics of the patients found in the current literature: age, sex (Male), BMI, CCI, CFS prior to hospital admission, BI prior to hospital admission, employment prior to hospital admission, ICU admission route of Emergency Room, source of Infection of abdomen, respiratory, urinary tract, musculoskeletal or soft tissue, SOFA sum score, septic shock, lactate level at the time of ICU admission, NPPV, HFNC, IMV, RRT, and length of hospital stay [29, 34–38]. In the multivariable analysis, the incidence of physical and cognitive dysfunction and mental disorders at the last follow-up were included as covariates. For instance, in the multivariable analysis examining the risk factors for physical dysfunction at the 12-month follow-up, the incidence of physical and cognitive dysfunction, along with mental disorders at the 6-month follow-up, was utilized as one of the covariates. To prevent overfitting in the multivariable analysis, we tested two additional models. The first model was analyzed using only the minimum variables, which included the incidence of physical and cognitive dysfunction, as well as mental disorders at the last follow-up. The second model included a small number of variables: Age, Sex, Body Mass Index, Clinical Frailty Scale, SOFA sum score, and the incidence of physical and cognitive dysfunction and mental disorders at the last follow-up. Missing data at each follow-up were not imputed in this analysis. All analyses were conducted using JMP software (version 13.0; SAS Institute Inc., Cary, NC, USA). Statistical tests were two-sided, and statistical significance was defined as a p value of less than 0.05.

## Results

### Patient characteristics

**Fig. 1:**
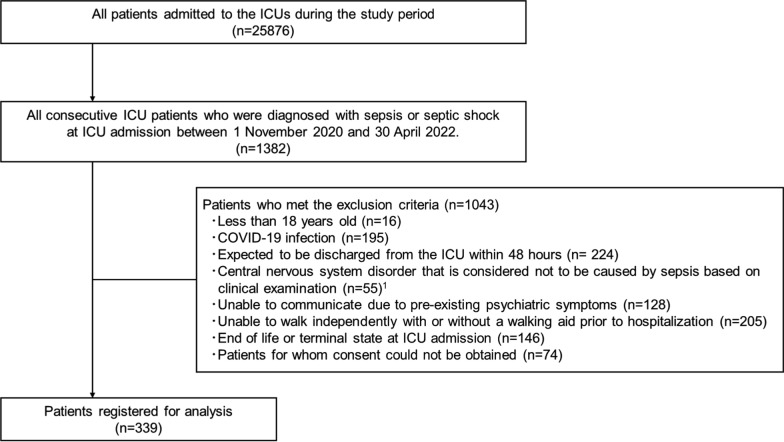
Patient flow chart. COVID-19: Coronavirus disease 2019, *ICU* Intensive Care Unit

Out of 25,876 ICU admissions, 1382 patients were diagnosed with sepsis or septic shock at ICU admission during the study period, and a total of 339 patients were registered for the analysis of this study (Fig. 1).

The patients were old (median age of 74 years old [IQR: 67–82]), predominantly male (60%), and low BMI of 23.4 kg/m^2^ [20.4–25.8]. They had few comorbidities (a median of 2 according to CCI), a median score of 3 in CFS [3–5], and a median of 100 in BI [90–100] prior to hospital admission. Around a quarter of patients (n = 80, 24%) were employed prior to hospital admission. The most common source of infection was the abdomen (32%), followed by the urinary tract (19%), musculoskeletal and soft tissue infections (19%), and respiratory infections (17%). At ICU admission, the SOFA score was a median of 9 [6–12] and septic shock was identified in 77%. Additionally, 61% and 40% of the patients underwent IMV and RRT during ICU stay, respectively. The median duration of IMV was 5.0 days [2.8–10.0], with median length of ICU and hospital stays being 6.1 days [3.7–10.8], and 28.7 days [16.5–51.5], respectively.

### Primary outcome

**Fig. 2:**
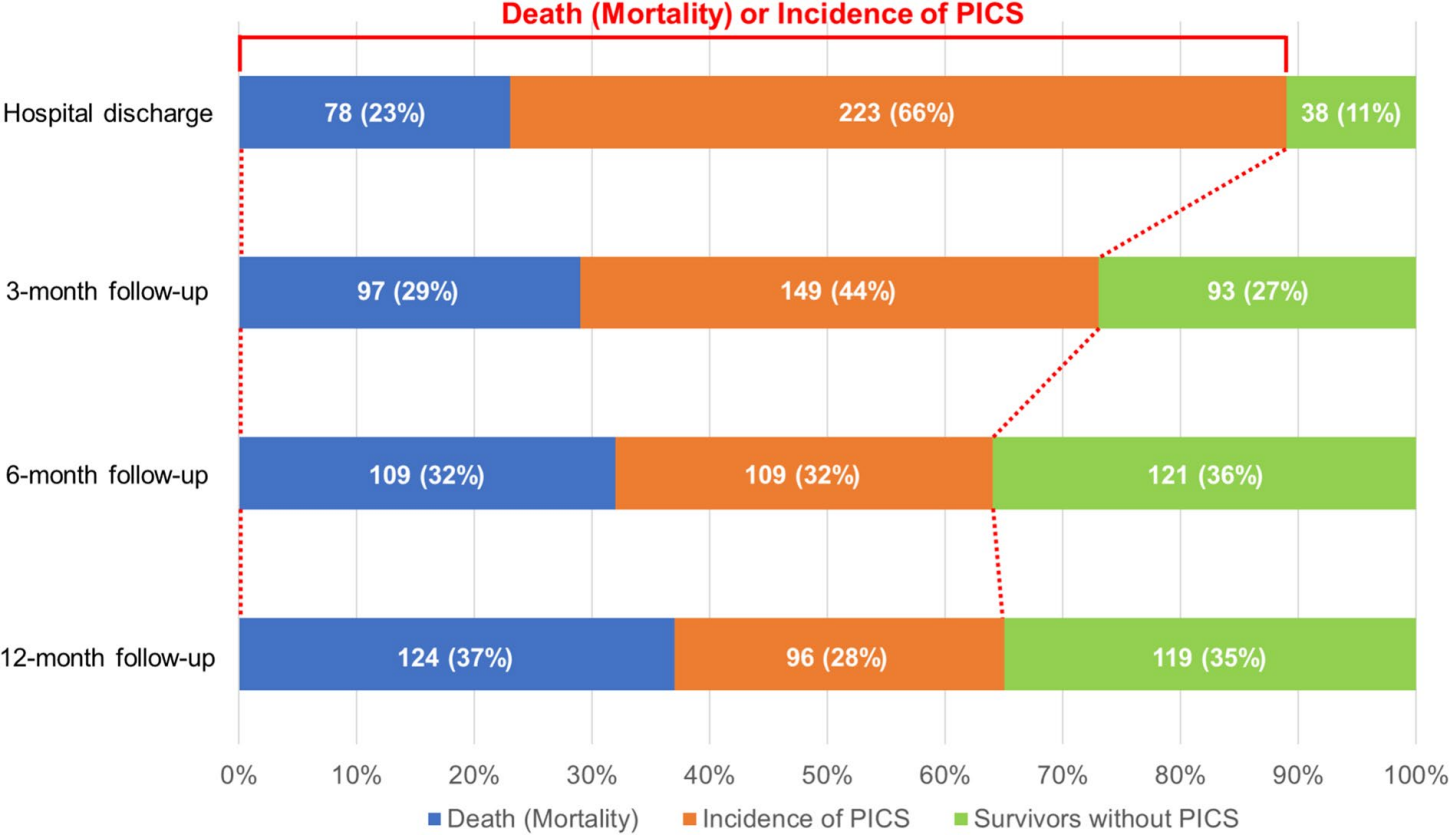
Primary outcome of the death or incidence of PICS. *PICS* Post Intensive Care Syndrome

The incidence of death or those who met the PICS Criteria at hospital discharge was 89%, with the combined mortality and PICS incidence, the primary outcome, of 73%, 64%, and 65%, at the 3-, 6-, and 12-month follow-up, respectively (Fig. 2). Mortality consistently increased from 23% at hospital discharge to 37% at the 12-month follow-up. The proportion of patients who met the pre-defined PICS criteria at hospital discharge was 66%, reducing to a rate of 28% at the 12-month follow-up. The loss to follow-up rate during the period is shown in Supplemental Table 1. There was no difference in the characteristics of the patients among those who were followed up at each follow-up time point.

**Table 1:** Characteristics of the patients

Variable	Overall (n = 339)
Baseline characteristics	
Age (year)	74 [67–82]
Sex (Male)	203 (60)
Body Mass Index (kg/m^2^)	23.4 [20.4–25.8]
Charlson Comorbidity Index	2 [1–3]
Clinical Frailty Scale prior to hospital admission	3 [3–5]
Barthel Index prior to hospital admission	100 [90–100]
Employment status prior to hospital admission	80 (24)
ICU admission route	
ER	246 (73)
General ward	85 (25)
Others	8 (2)
Source of infection	
Abdomen	111 (32)
Urinary tract	63 (19)
Musculoskeletal and soft tissue	63 (19)
Respiratory	57 (17)
Other	45 (13)
Sequential Organ Failure Assessment (SOFA) sum score	9 [6–12]
Lactate level at ICU admission (mmol/L)	3.1 [1.6–5.8]
Presence of septic shock at ICU admission	261 (77)
Use of vasoactive drugs during ICU stay	
Noradrenaline	298 (88)
Vasopressin	145 (43)
Epinephrine	42 (12)
Use of medical devices during ICU stay	
Noninvasive positive pressure ventilation (NPPV)	27 (8)
High flow nasal cannula (HFNC)	57 (17)
Invasive mechanical ventilation (IMV)	208 (61)
Renal replacement therapy (RRT)	136 (40)
Clinical consequences of hospital stay	
ICU mortality	39 (12)
Duration of invasive mechanical ventilation	5.0 [2.8–10.0]
Length of ICU stay (days)	6.1 [3.7–10.8]
Length of Hospital stay (days)	28.7 [16.5–51.5]
Number of patients who met PICS criteria at hospital discharge	
Incidence of PICS	223 (66)
Incidence of physical dysfunction	164 (48)
Incidence of cognitive dysfunction	145 (43)
Incidence of mental disorder	129 (38)

The data in the table are presented as median with inter quartile range [IQR] or number with percentage (%)

*ICU* Intensive Care Unit, *PICS* Post-Intensive Care Syndrome

### Outcomes among survivors

Among the survivors discharged from hospital, 85% met the criteria for PICS at the point of hospital discharge (Table 2). This reduced over the post-discharge period with reported rates of PICS as 62%, 47%, and 45% at the 3-, 6- and 12-month follow-ups. All three domains of PICS, including physical and cognitive dysfunction as well as mental disorders, consistently decreased during the first year of survivorship. Among the three symptoms of mental disorders, depression was consistently more prevalent over the year compared to anxiety or PTSD. The patients often experienced two or more dysfunctions simultaneously, decreasing from 59% of survivors at hospital discharge to 21% at the 12-month follow-up. The translated index value of EQ-5D-5L slightly improved over the year, while the EQ-VAS, a self-reported QOL value, showed less improvement. The overall employment status remained low over the 12 months following the discharge (15–17%), with slight reductions seen by 12 months in those survivors who had a job prior to hospitalization (52% at 3 months down to 44% at 12 months). Body weight was found to have decreased from baseline at the point of hospital discharge. Although gradual improvement was seen over the year following discharge from the hospital, body weight failed to return to baseline levels before the sepsis-related hospitalization. Two-fifths were readmitted to the hospital and one tenth were readmitted to ICU within the first year after discharge, and nearly one-third of the survivors utilized emergency services. The use of physical rehabilitation or psychiatric consultation clinics remained consistently low during the first year, not only among all survivors but also among those who experienced physical dysfunction or mental disorders.

**Table 2:** Outcomes among survivors

Item	Overall (n = 339)			
	Hospital discharge (n = 261)	3 month (n = 242)	6 month (n = 230)	12 month (n = 215)
Incidence of PICS among survivors	223 (85)	149 (62)	109 (47)	96 (45)
Incidence of physical dysfunction	164 (63)	79 (32)	55 (24)	42 (20)
A Score of BI	80 [40–100]	100 [75–100]	100 [85–100]	100 [90–100]
Incidence of cognitive dysfunction	145 (56)	85 (35)	57 (25)	63 (29)
A Score of SMQ	37 [27–42]	40 [33–45]	41 [36–45]	42 [36–45]
Incidence of mental disorder	129 (49)	77 (32)	68 (30)	50 (23)
Incidence of anxiety symptom	49 (19)	34 (14)	34 (15)	21 (10)
A score in the subscale for anxiety of HADS	4 [2–7]	4 [1–7]	3 [1–7]	2 [1–6]
Incidence of depression symptom	107 (41)	60 (25)	60 (26)	44 (20)
A score in the subscale for depression of HADS	7 [4–11]	5 [2–8]	5 [2–9]	5 [2–8]
Incidence of PTSD symptom	24 (9)	29 (12)	25 (11)	15 (7)
A Score of IES-R	4 [2–14]	7 [2–20]	7 [1–18]	6 [1–14]
Patients with two of three dysfunctions	81 (31)	39 (16)	25 (11)	30 (14)
Patients with all three domains of dysfunctions	72 (28)	27 (11)	23 (10)	15 (7)
EQ-5D-5L translated value	0.69 [0.42–0.83]	0.78 [0.55–0.89]	0.78 [0.60–0.89]	0.82 [0.68–1.00]
EQ-VAS	50 [7–70]	50 [10–80]	60 [8–80]	60 [30–80]
Employment status		36 (15)	34 (15)	37 (17)
Employment among those who had a job prior to the hospitalization^a^		34/66 (52)	30/66 (45)	27/62 (44)
Body weight (kg)		56 [47–64]	56 [49–65]	57 [48–66]
Changes in body weight as compared to the baseline (kg)		−4.0 [−0.6 ~ −8.9]	−3.2 [−8.1 ~ 0]	−2.0 [−6.0 ~ 1.4]
Changes in body weight as compared to the baseline (%)		−6.5 [−13.5 ~ −0.8]	−5.1 [−13.35 ~ 0]	−2.9 [−9.7 ~ 2.2]
Readmission to hospital after discharge		56 (24)	60 (28)	78 (41)
Readmission to ICU after discharge		8 (3)	12 (6)	16 (9)
Unplanned emergency room visits		19 (8)	37 (17)	58 (31)
Utilization of the physical rehabilitation clinic		20 (9)	30 (14)	28 (15)
Utilization of the physical rehabilitation clinic among those with physical dysfunction^b^		8/79 (10)	8/55 (15)	12/42 (29)
Utilization of the psychiatric consultation clinic		11 (5)	15 (7)	14 (7)
Utilization of the psychiatric consultation clinic among those with mental disorders^c^		9/77 (12)	8/68 (12)	7/50 (14)

Data in the table are presented as median with Inter Quartile Range (IQR) or number with percentage

*BI* Barthel Index, *HADs* Hospital Anxiety and Depression Scale, *ICU* Intensive Care Unit, *IES-R* Impact of Events Scale-Revised, *EQ-5D-5L* EuroQoL 5-dimension 5-level, *PICS* Post-Intensive Care Syndrome, *SMQ* Short Memory Questionnaire, *VAS* Visual Analogue Scale

^a^Denominators represent the number of survivors who had a job prior to the hospitalization at the time of follow-ups

^b^Denominators represent the number of survivors who had physical dysfunction at the time of follow-ups

^c^Denominators represent the number of survivors who had mental disorders at the time of follow-ups

### Risk factors

The incidence of physical, cognitive, or mental dysfunction was consistently and significantly associated with the incidence of the same dysfunction at the next follow-up (Table 3). This result remained consistent when the variables in the multivariable analysis were changed (Supplemental Table 2 and 3). Among the factors included in the multivariable analysis, age was consistently identified as an independent factor for physical and cognitive dysfunctions, and HFNC was for mental disorders (Supplemental Tables 4, 5, and 6).

**Table 3:** Association of PICS at a follow-up with its prior follow-up

Variable	Physical dysfunction					
	3-month		6-month		12-month	
	Odds ratio	P value	Odds ratio	P value	Odds Ratio	P value
Incidence of physical dysfunction at the previous follow-up	7.21 [2.21–23.53]	< 0.01	24.24 [6.11–96.20]	< 0.01	21.41 [3.67–124.85]	< 0.01
Incidence of cognitive dysfunction at the previous follow-up	1.22 [0.41–3.65]	0.72	0.78 [0.20–3.04]	0.73	1.22 [0.16–9.07]	0.85
Incidence of mental disorder at the previous follow-up	1.42 [0.57–3.54]	0.46	3.15 [0.75–13.26]	0.12	1.12 [0.19–6.55]	0.90

Variable	Cognitive dysfunction					
	3-month		6-month		12-month	
	Odds ratio	P value	Odds ratio	P value	Odds Ratio	P value
Incidence of physical dysfunction at the previous follow-up	1.31 [0.53–3.23]	0.56	5.19 [1.31–20.61]	0.02	0.41 [0.10–1.65]	0.21
Incidence of cognitive dysfunction at the previous follow-up	3.97 [1.59–9.86]	< 0.01	16.29 [4.39–60.41]	< 0.01	4.12 [1.37–12.41]	0.01
Incidence of mental disorder at the previous follow-up	1.08 [0.48–2.41]	0.86	3.83 [1.05–14.02]	0.04	1.11 [0.37–3.30]	0.86

Variable	Mental disorders					
	3-month		6-month		12-month	
	Odds ratio	P value	Odds ratio	P value	Odds Ratio	P value
Incidence of physical dysfunction at the previous follow-up	2.29 [0.84–6.21]	0.11	2.09 [0.68–6.40]	0.20	0.95 [0.23–3.95]	0.95
Incidence of cognitive dysfunction at the previous follow-up	1.77 [0.69–4.55]	0.24	0.69 [0.25–1.88]	0.46	4.23 [1.24–14.44]	0.02
Incidence of mental disorder at the previous follow-up	4.30 [1.77–10.42]	< 0.01	10.37 [3.59–29.97]	< 0.01	7.18 [2.21–23.34]	< 0.01

The data in the table are presented as odds ratio with 95% confidence interval

## Discussion

This multicenter prospective cohort study, conducted at 21 ICUs in 20 tertiary hospitals in Japan, demonstrated the characteristics of ICU patients with sepsis, the trajectories of their prognostic and functional outcomes (as a composite outcome of death or the incidence of PICS), and the impact of hospitalization on their post-sepsis lives over the first year after hospital discharge.

The results indicated that the patient characteristics were significantly different from those reported in previous studies of sepsis outside of Japan, highlighting the challenges of translating the past findings to clinical practice in Japan and underscoring the urgent need for studies conducted in Japan. Surprisingly, only one-third of ICU patients with sepsis survived without PICS, even a year after being discharged from the hospital. Furthermore, this situation did not improve between 6 and 12 months, indicating recovery from functional dysfunctions could reach a plateau within the first six months after hospital discharge.

The patients enrolled in this study were relatively older and had a lower BMI in comparison to previous sepsis studies, particularly those conducted in Western countries (Supplemental Table 7) [12, 13, 37, 39, 40]. Both advanced age and lower BMI independently correlate with long-term outcomes, including mortality and functional outcomes such as physical strength [41–45]. In addition, the patient cohort in this study was already on the brink of frailty based on the CFS value (a median of 3 [IQR: 3–5]) at ICU admission. This is likely due to the older age of our study population, which also serves as an independent factor affecting long-term outcomes [41, 46]. The source of infection was also different from sepsis-related studies conducted in Western countries, potentially due to the geographical or cultural background difference. These differences need to be taken into account when developing tailored interventions for the sepsis patient cohort in Japan [47]. Recent literature emphasizes that the variability and heterogeneity in patient cohorts highlight the necessity for personalized approaches that consider individual patient profiles, rather than adopting an one-size-fits-all strategy [6, 36, 48]. This, however, can be also an alert when implementing evidence-based guidelines that were based on the findings from a patient cohort with distinct characteristics [49]. To promote a deeper understanding of long-term outcomes in the sepsis patient cohort in Japan and enhance their future care, a large-scale cohort registry with a comprehensive follow-up system is essential. In the future, Assistive Artificial Intelligence or robust machine learning methods might help identify patients at high risk of developing PICS and ultimately help to direct the prompt delivery of tailored interventions according to their risk assessment [50]. Currently, this study is ongoing to collect long-term outcomes for up to five years post-hospital discharge [19].

This study also revealed that a significant portion of ICU patients with sepsis either died or experienced serious functional deterioration which persisted for one year after hospital discharge. Only one-third of sepsis survivors were able to survive without PICS. Recovery appeared to plateau at six months after discharge, with no further significant change observed between six and twelve months, which is consistent with the recent report in the ICU population with COVID-19 infection in Japan [34]. Regarding the utilization of healthcare resources and services, a significant demand for resource utilization was evident, with a high rate of readmissions to the hospital or ICU, as well as visits to the emergency department. There was low utilization of healthcare services such as physical rehabilitation or psychiatric consultation clinics, even among those experiencing physical dysfunction or mental disorders. Given the substantially increasing burden on the healthcare system in terms of financial costs and the growing number of patients who continue to suffer after a critical illness [6], this imbalance urgently needs to be addressed through adequate support and appropriate resource allocation. As discussed that the overall incidence of PICS plateaued between 6 and 12 months, it is important to consider effective and prompt interventions soon or at least in the first 6 months after hospital discharge, not to miss the potentially critical period for recovery. Such actions will be crucial in facilitating survivors’ return to their lives and reducing the healthcare burden. This is further supported by the finding that PICS-related dysfunctions independently contributed to the experience of PICS in the following three to six months. [6, 58, 59]. Investigating the effects of developed interventions in an appropriate manner, such as randomization, will be crucial to accelerate the flow toward their implementation in clinical settings. Future studies are also needed to verify whether developed interventions can reduce healthcare resource usage and readmissions.

In this study, only small improvements were observed in QOL despite the almost halved incidence rate of PICS-associated dysfunctions among survivors. As shown in earlier studies, an improvement in symptoms associated with PICS did not lead to significant improvements in QOL scores (EQ-5D-5L) or self-reported QOL (EQ-VAS), suggesting that evaluating PICS-associated symptoms only is likely to be insufficient to assess their post-illness life [51, 52]. QOL could recover when other symptoms (i.e., PICS-associated dysfunctions) were first addressed, suggesting the last indicator to reflect overall recovery from sepsis. Therefore, QOL should be considered alongside assessments of PICS in future studies [53]. In this study, only half of those who were employed prior to hospitalization returned to work at three months, and around 20% of them lost their jobs between three and twelve months after discharge. This outcome was worse than earlier reports from Japan involving a general ICU patient cohort, where 20% of employed patients became unemployed by the twelve-month follow-up [54]. This may be due to sepsis resulting in a more challenging post-illness life than other ICU diseases because of its severity [13, 55]. Although our results do not clarify the relationship between job loss and PICS, this underscores the need for additional support for ICU patients with sepsis to facilitate their return to and maintenance of employment [56, 57]. Further research, including qualitative interview surveys, would aid in understanding the key reasons behind job loss in the post-sepsis phase. Interestingly, the use of HFNC was associated with mental disorders. This may be, as previous reports suggested, because patients receiving HFNC were conscious during all medical events or procedures, which could be harmful, delusional, or lead to distorted memories, resulting in long-term health issues [60, 61].

Several limitations were acknowledged. First, the generalizability of this study requires attention when interpreting the results. This study involved a representative sample of 21 ICUs from 20 tertiary hospitals in Japan, covering only 7% of the ICU beds in the country. Given that the prevalence of sepsis in this study (5.3% of all ICU admissions) was similar to that in the previous cohort registry of ICU patients in Japan (4.3%) [14, 62], the findings of this study could have potential generalizability across Japan. Furthermore, the strength of this study lies in its exclusive focus on sepsis patients in the ICU, whereas the recent PICS study in Japan included a general ICU population, encompassing trauma and burn cases [28]. These cases tend to have a significantly different trajectory of functional recovery due to their specific disease nature and the procedures involved. The high follow-up rate should also be highlighted as the strength of this study, which could minimize the selection bias during the follow-up period. However, the exclusion of a large number of patients also needs to be taken into account. For example, the patients included in this study showed a low frequency of pulmonary infections, which was typically and frequently reported in the ICU population in Japan [63]. Second, the definition of PICS may vary between studies [27]. For example, while some prior studies have also employed the BI to assess physical function, others used different assessment tools. As a result, the incidence of PICS could differ when different assessment tools are applied. The results could also be biased by different assessors, especially when involving a large number of hospitals. Ideally, the outcome measurement should be performed by someone who is not involved in ICU care and not in a relationship with the patient or families to obtain objective outcomes. Nonetheless, the strength of this study lies in capturing a wide range of post-sepsis life, not only PICS-associated outcomes but also QOL, employment status after hospital discharge, and the utilization of healthcare resources and services. Third, the risk factors for PICS at each follow-up time point were identified in the multivariable analysis, although the causal relationship cannot be discussed within the constraints of this study’s design. Furthermore, potential independent factors like prior psychiatric illness and social factors (i.e., family support status, economic conditions, etc.) were not collected in this study. Fourth, this study was conducted during the COVID-19 pandemic, a time when hospitals and ICUs were significantly impacted. Thus, the current situation may differ from when this study was conducted, possibly resulting in a different post-sepsis life. To address these limitations, we propose a large-scale cohort registry of ICU sepsis in Japan with regular follow-up systems to comprehend the trajectories of post-sepsis life and develop effective interventions.

## Conclusions

This multicenter prospective cohort study revealed the reality of post-sepsis recovery in ICU patients in Japan, who had a significantly different patient profile compared to previous PICS-related studies. Our results indicate major challenges exist in supporting the recovery of functions and improving the return to daily life in survivors of sepsis.

## Supplementary Information

Additional file 1

Additional file 2. Table 1: Follow-up loss rate at each time point. Table 2. Association of PICS at a follow-up with its prior follow-up in the multivariable analysis with the minimum variable. Table 3. Association of PICS at a follow-up with its prior follow-up in the multivariable analysis with a few variables. Table 4. Risk factors for the incidence of physical dysfunction at the time of each follow-up. Table 5. Risk factors for the incidence of cognitive dysfunction at the time of each follow-up. Table 6. Risk factors for the incidence of mental disorders at the time of each follow-up. Table 7. Differences in patient characteristics from past papers

## Data Availability

No datasets were generated or analysed during the current study.

## Abbreviations

ADL: Activities of daily living
BI: Barthel index
BMI: Body mass index
CCI: Charlson comorbidity index
CFS: Clinical frailty scale
COVID-19: Coronavirus disease 2019
EQ-5D-5L: EuroQoL 5-dimension 5-level
EQ-VAS: EuroQoL-visual analog scale
HADS: Hospital anxiety and depression scale
HFNC: High-flow nasal cannula
ICU: Intensive care unit
IES-R: Impact of events scale-revised
IMV: Invasive mechanical ventilation
IQR: Interquartile range
J-SSCG: Japanese clinical practice guidelines for management of sepsis and septic shock
NPPV: Noninvasive positive pressure ventilation
PICS: Post-intensive care syndrome
QOL: Quality of Life
RRT: Renal replacement therapy
SMQ: Short memory questionnaire
SOFA: Sequential organ failure assessment
STROBE: Strengthening the reporting of observational studies in epidemiology
UMIN: University hospital medical information network
