# Correction: One-year outcomes in sepsis: a prospective multicenter cohort study in Japan

**DOI:** 10.1186/s40560-025-00799-7

**Published:** 2025-05-22

**Authors:** Keibun Liu, Shinichi Watanabe, Kensuke Nakamura, Hidehiko Nakano, Maiko Motoki, Hiroshi Kamijo, Matsuoka Ayaka, Kenzo Ishii, Yasunari Morita, Takashi Hongo, Nobutake Shimojo, Yukiko Tanaka, Manabu Hanazawa, Tomohiro Hamagami, Kenji Oike, Daisuke Kasugai, Yutaka Sakuda, Yuhei Irie, Masakazu Nitta, Kazuki Akieda, Daigo Shimakura, Hajime Katsukawa, Toru Kotani, David McWilliams, Peter Nydahl, Stefan J. Schaller, Takayuki Ogura, the ILOSS Study Group

**Affiliations:** 1 Non-Profit Organization ICU Collaboration Network (ICON), Tokyo, Japan; 2 Department of Physical Therapy, Gifu University of Health Science, Gifu, Japan; 3 Department of Rehabilitation, National Hospital Organization Nagoya Medical Center, Nagoya, Aichi Japan; 4 Department of Critical Care Medicine, Yokohama City University Hospital, 3-9, Fukuura, Kanazawa-Ku, Yokohama, Kanagawa 236-0004 Japan; 5 Department of Emergency and Critical Care Medicine, Hitachi General Hospital, Hitachi, Ibaraki Japan; 6 Department of Emergency and Critical Care Medicine, Shinshu University School of Medicine, Nagano, Japan; 7 Department of Emergency and Critical Care Medicine Faculty, Saga University Hospital, Saga, Saga Japan; 8 Department of Anesthesiology, Intensive Care Unit, Fukuyama City Hospital, Fukuyama, Hiroshima Japan; 9 Department of Emergency and Intensive Care Medicine, National Hospital Organization Nagoya Medical Center, Nagoya, Aichi Japan; 10 Department of Emergency, Critical Care, and Disaster Medicine, Okayama University Graduate School of Medicine, Dentistry, and Pharmaceutical Sciences, 2-5-1 Shikata-Cho, Okayama Kita-Ku, Okayama, 700-8558 Japan; 11 Department of Emergency, Okayama Saiseikai General Hospital, 2-25 Kokutaityo, Okayama Kita-Ku, Okayama, 700-8511 Japan; 12 Emergency and Critical Care Medicine, Faculty of Medicine, University of Tsukuba, Ibaraki, Japan; 13 Department of Emergency, Tsukuba Medical Center Hospital, Tsukuba, Ibaraki Japan; 14 Department of Rehabilitation, Japan Red Cross Narita Hospital, Narita, Chiba Japan; 15 Tajima Emergency & Critical Care Medical Center, Toyooka Public Hospital, Toyooka, Hyogo Japan; 16 Department of Rehabilitation, Tsuchiura Kyodo General Hospital, Tsuchiura, Ibaraki Japan; 17 Department of Emergency and Critical Care Medicine, Nagoya University Graduate School of Medicine, Nagoya, Aichi Japan; 18 Department of Intensive Care Medicine, Okinawa Kyodo Hospital, Naha, Okinawa Japan; 19 Department of Emergency and Critical Care Medicine, Fukuoka University Hospital, Fukuoka, Fukuoka Japan; 20 Department of Intensive Care Unit, Niigata University Medical and Dental Hospital, Niigata, Niigata, Japan; 21 Department of Emergency Medicine, SUBARU Health Insurance Society Ota Memorial Hospital, Ota, Gunma Japan; 22 Graduate School of Data Science, Shiga University, Shiga, Japan; 23 Japanese Society for Early Mobilization, Tokyo, Japan; 24 Showa Medical University, Shinagawa, Tokyo Japan; 25 Centre for Care Excellence, Coventry University, Coventry, UK; 26 Critical Care, University Hospitals Coventry & Warwickshire NHS Trust, Coventry, UK; 27 Nursing Research, University Hospital Schleswig-Holstein, Kiel, Germany; 28 Institute of Nursing Science and Development, Paracelsus Medical University, Salzburg, Austria; 29 Department of Anesthesia, Intensive Care Medicine and Pain Medicine, Clinical Division of General Anesthesia and Intensive Care Medicine, Medical University of Vienna, Wien, Austria; 30 Department of Anesthesiology and Intensive Care Medicine (CCM/CVK), Charité - Universitätsmedizin, Berlin, Germany; 31 Department of Emergency Medicine and Critical Care Medicine, Tochigi Prefectural Emergency and Critical Care Center, Saiseikai Utsunomiya Hospital, Utsunomiya, Tochigi Japan; 32 Present Address: 2-15-13 Hongo, Bunkyo-Ku, Tokyo, 113-0033 Japan

**Correction:** **Journal of Intensive Care (2025) 13:23** 10.1186/s40560-025-00792-0

Following publication of the original article [1], a sentence in Abstract Result section has been corrected from: “The rate of death **for** those who met PICS Criteria at hospital discharge was 89%, with a death or PICS incidence of 73%, 64%, and 65% at 3, 6, and 12 months, respectively.”

to: “The rate of death **or** those who met PICS Criteria at hospital discharge was 89%, with a death or PICS incidence of 73%, 64%, and 65% at 3, 6, and 12 months, respectively.”

The original article [1] has been updated.
