# Supplementary material for: One-year outcomes in sepsis: a prospective multicenter cohort study in Japan
Source: J Intensive Care. 2025 May 1;13:23. doi: 10.1186/s40560-025-00792-0 (PMC12044722; doi:10.1186/s40560-025-00792-0)
Supplement: Supplementary file 1 — Additional file 1 [file 40560_2025_792_MOESM1_ESM.docx]

Appendix 1: ILOSS Study Team-2024-03-30

**LIST OF COLLABORATORS**

| HOSPITAL | COLLABORATORS |
| --- | --- |
| Saiseikai Utsunomiya Hospital (7) | Takayuki Ogura  Chihiro Takai  Sou Yamada  Yusuke Minagawa  Nao Takano  Kyohei Ishikawa |
| Showa University School of Medicine (6) | Toru Kotani  Maiko Mori  Fumihito Kasai  Hiroko Maruo  Kota Kubodera  Kaori Tsuruta |
| Hitachi General Hospital (2) | Kensuke Nakamura  Hidehiko Nakano |
| National Hospital Organization Nagoya Medical Center (3) | Shinichi Watanabe  Mika Ohno  Yayoi Honjyo |
| Fukuyama City Hospital (7) | Kenzo Ishii  Shuhei Ikeguchi  Yoshiyuki Teranobu  Shogo Akamatsu  Yuichi Yoshioka  Takuya Okuda  Suzuka Sato |
| Japan Red Cross Maebashi Hospital (5) | Kenji Fujizuka  Akira Kawauchi  Takeshi Mizuno  Kei Tsunoda  Emi Abe |
| Naha City Hospital (8) | Yasumura Daisetsu  Kawabata Shinya  Tonaki Takuya  Miyagi　Yuuichi  Kamiya Taisuke  Touyama　Hiroyuki  Miyata Yuuji  Tomiyama Hiroshi |
| Toyooka Public Hospital (15) | Daisuke Taniguchi  Tomohiro Hamagami  Hiroaki Hanafusa  Hiroki Kaya  Yoshiko Hayashi  Toshiki Fujii  Tomoyuki Ishida  Akari Kohama  Yui Ezaki  Yoichiro Yamasaki  Kazuki Yamada  Masaomi Tamura  Chinatsu Yoneda  Shohei Nishida  Mitsuaki Ihara |
| University of Tsukuba Hospital (4) | Nobutake Shimojo  Yoshiaki Inoue  Yuki Enomoto  Shizuko Gomi |
| Niigata University Medical and Dental hospital (4) | Masakazu Nitta  Tadayuki Honda  Natsuo Kamimura  Taro Tamakawa |
| Okayama Saiseikai General Hospital (3) | Hongo Takashi  Nozaki Satoshi  Mizukawa Syunichi |
| SUBARU Health Insurance Sociaty Ota Memorial Hospital (8) | Kazuki Akieda  Shohei Matsumoto  Takeshi Nara  Keiji Sakurai  Hideaki Kanazashi  Yohei Tsubouchi  Junya Matsushima  Takayuki Yasuoka |
| Showa University Fujigaoka Hospital (8) | Maeda Atsuo  Hayashi Munetaka  Sasaki　Jun  Ohno Takanori  Nakajima Yasuhiro  Harano Kouhei  Sugimoto Tatsuya  Hiruma Kaeda |
| Mie University Hospital (2) | Tadashi Kaneko  Susumu Nakahashi |
| Okinawa Kyodo Hospital (6) | Yutaka Sakuda  Kiyohiko Kinjoh  Takayuki Yonaha  Kyousuke Taira  Gennki Ishigaki  Katuya Matubara |
| Sendai City Hospital (10) | Kohkichi Andoh  Yosinobu Kameyama  Yumiko Sakurada  Koko Adachi  Yutaro Funahashi  Satoshi Yamanouchi  Yuji Murata  Yusuke Konta  Keishi Takase  Kaoru Onoyama |
| St. Marianna University, School of Medicine, Yokohama-city Seibu Hospital (2) | Akiyoshi Nagatomi  Shinya Matsushima |
| Tsukuba Medical Center Hospital (15) | Yukiko Tanaka  Hisako Saito  Hiroki Namekawa  Ryota Watahiki  Momoko Konishi  Akemi Fujita  Hiroko Hirose  Sayaka Tsukamoto  Kyoko Shibata  Kumiko kaitsuka  Rie Awano  Masami Okubo  Tomoya Morita  Ikuo Aita  Mototsugu Kohno |
| Fukuoka University Hospital (7) | Yuhei Irie  Yoshihiko Nakamura  Reiko Yamasaski  Shinichi Morimoto  Yoshito Izutani  Maiko Nakashio  Aya yastugi |
| Shinshu University Hospital (11) | Hiroshi Kamijo  Katsunori Mochizuki  Keisuke Iida  Takuya Kishida  Hiroto Sakamoto  Yasuaki Maeda  Masaya Joko  Shota Tsukakoshi  Yukako Yamazaki  Yasunari Sakai  Shuhei Yamamoto |
| Japan Red Cross Narita Hospital (5) | Manabu Hanazawa  Yoshihisa Tateishi  Ayaka Baba  Kiyoko Iida  Shinji Kishimoto |
| Nagoya University Graduate School of Medicine (11) | Daisuke Kasugai  Akiko Takaoka  Hitomi Kokubo  Tomoko Inaba  Kaori Koga  Koto Yamazaki  Mai Yamashita  Miho Shimizu  Yohei Tsuchikawa  Shinya Tanaka  Hiromasa Yamamoto |
| Saga University Hospital (6) | Matsuoka Ayaka  Sakamoto Yuichirou  Mouri Kousuke  Komaki Moe  Higuchi Masahiro  Yamazaki Hirotaka |
| Tsuchiura Kyodo General Hospital (5) | Kenji Oike  Yuuichi Araki  Naoki Taka  Kenichi Igashira  Osamu Ishibashi |
