# Supplementary material for: One-year outcomes in sepsis: a prospective multicenter cohort study in Japan
Source: J Intensive Care. 2025 May 1;13:23. doi: 10.1186/s40560-025-00792-0 (PMC12044722; doi:10.1186/s40560-025-00792-0)
Supplement: Supplementary file 2 — Additional file 2. Table 1: Follow-up loss rate at each time point. Table 2. Association of PICS at a follow-up with its prior follow-up in the multivariable analysis with the minimum variable. Table 3. Association of PICS at a follow-up with its prior follow-up in the multivariable analysis with a few variables. Table 4. Risk factors for the incidence of physical dysfunction at the time of each follow-up. Table 5. Risk factors for the incidence of cognitive dysfunction at the time of each follow-up. Table 6. Risk factors for the incidence of mental disorders at the time of each follow-up. Table 7. Differences in patient characteristics from past papers [file 40560_2025_792_MOESM2_ESM.docx]

**Online Supplemental Materials**

Supplemental Table 1: Follow-up loss rate at each time point

Supplemental Table 2: Characteristics of the patients who were followed-up at each follow-up

Supplemental Table 3. Association of PICS at a follow-up with its prior follow-up in the multivariable analysis with the minimum variable

Supplemental Table 4. Association of PICS at a follow-up with its prior follow-up in the multivariable analysis with a few variables

Supplemental Table 5. Risk factors for the incidence of physical dysfunction at the time of each follow-up

Supplemental Table 6. Risk factors for the incidence of cognitive dysfunction at the time of each follow-up

Supplemental Table 7. Risk factors for the incidence of mental disorders at the time of each follow-up

Supplemental Table 8. Differences in patient characteristics from past papers

**Supplemental Table 1: Follow-up loss rate at each time point**

|  | Overall patients registered (n=339) | | | |
| --- | --- | --- | --- | --- |
|  | Hospital discharge | 3 months | 6 months | 12 months |
| Overall follow-up loss | 0 (0%) | 8 (2) | 14 (4) | 27 (8) |

Data in the table are presented as number with percentage.

**Supplemental Table 2: Characteristics of the patients who were followed-up at each follow-up**

| Variable | Overall (n=339) | 3 months (n=331) | 6 months (n=325) | 12 months (n=312) |
| --- | --- | --- | --- | --- |
| Baseline characteristics |  |  |  |  |
| Age (year) | 74 [67-82] | 74 [67-82] | 74 [67-82] | 74 [66-82] |
| Sex (Male) | 203 (60) | 196 (59) | 191 (59) | 182 (58) |
| Body Mass Index (kg/m^2^) | 23.4 [20.4-25.8] | 23.4 [20.4-25.8] | 23.4 [20.6-25.8] | 23.4 [20.4-25.8] |
| Charlson Comorbidity Index | 2 [1-3] | 2 [1-3] | 2 [1-3] | 2 [1-2] |
| Clinical Frailty Scale prior to hospital admission | 3 [3-5] | 3 [3-5] | 3 [3-5] | 3 [3-5] |
| Barthel Index prior to hospital admission | 100 [90-100] | 100 [90-100] | 100 [90-100] | 100 [90-100] |
| Employment status prior to hospital admission | 80 (24) | 79 (24) | 79 (24) | 74 (23) |
| ICU admission Route |  |  |  |  |
| ER | 246 (73) | 240 (73) | 236 (73) | 230 (73) |
| General ward | 85 (25) | 83 (25) | 81 (25) | 75 (24) |
| Others | 8 (2) | 8 (2) | 8 (2) | 7 (3) |
| Source of Infection |  |  |  |  |
| Abdomen | 111 (32) | 107 (32) | 106 (33) | 101 (32) |
| Urinary tract | 63 (19) | 61 (18) | 58 (18) | 58 (19) |
| Musculoskeletal and Soft tissue | 63 (19) | 63 (19) | 62 (19) | 60 (19) |
| Respiratory | 57 (17) | 56 (17) | 55 (17) | 52 (17) |
| Other | 45 (13) | 44 (14) | 44 (13) | 41 (13) |
| Sequential Organ Failure Assessment (SOFA) sum score | 9 [6-12] | 9 [6-12] | 9 [6-12] | 9 [6-12] |
| Lactate level at ICU admission (mmol/L) | 3.1 [1.6-5.8] | 3.1 [1.6-5.7] | 3.1 [1.7-5.7] | 3.1 [1.7-5.7] |
| Presence of Septic shock at ICU admission | 261 (77) | 255 (77) | 251 (77) | 242 (78) |
| Use of vasoactive drugs during ICU stay |  |  |  |  |
| Noradrenaline | 298 (88) | 290 (88) | 286 (88) | 274 (88) |
| Vasopressin | 145 (43) | 138 (42) | 135 (42) | 132 (42) |
| Epinephrine | 42 (12) | 41 (12) | 40 (12) | 40 (13) |
| Use of medical devices during ICU stay |  |  |  |  |
| Noninvasive positive pressure ventilation (NPPV) | 27 (8) | 26 (8) | 26 (8) | 24 (8) |
| High flow nasal cannula (HFNC) | 57 (17) | 54 (16) | 54 (16) | 49 (16) |
| Invasive mechanical ventilation (IMV) | 208 (61) | 203 (61) | 201 (62) | 193 (62) |
| Renal replacement therapy (RRT) | 136 (40) | 133 (40) | 132 (41) | 127 (41) |
| Clinical consequences of hospital stay |  |  |  |  |
| ICU mortality | 39 (12) | 39 (12) | 39 (12) | 39 (13) |
| Duration of invasive mechanical ventilation | 5.0 [2.8-10.0] | 5.0 [2.8-10.2] | 5.0 [2.8-10.1] | 5.0 [2.8-10.0] |
| Length of ICU stay (days) | 6.1 [3.7-10.8] | 6.1 [3.7-10.9] | 6.0 [3.7-10.8] | 6.0 [3.7-10.7] |
| Length of Hospital stay (days) | 28.7 [16.5-51.5] | 28.7 [16.5-51.1] | 28.1 [16.4-51.0] | 27.6 [16.3-49.0] |
| Number of patients who met PICS criteria at hospital discharge |  |  |  |  |
| Incidence of PICS | 223 (66) | 218 (66) | 212 (65) | 201 (64) |
| Incidence of physical dysfunction | 164 (48) | 161 (49) | 156 (48) | 149 (48) |
| Incidence of cognitive dysfunction | 145 (43) | 144 (44) | 139 (43) | 132 (42) |
| Incidence of mental disorder | 129 (38) | 127 (38) | 124 (38) | 115 (37) |

Data in the table are presented as median with Inter Quartile Range [IQR] or number with percentage (%).

ICU: Intensive Care Unit, PICS: Post Intensive Care Syndrome

**Supplemental Table 3. Association of PICS at a follow-up with its prior follow-up in the multivariable analysis with the minimum variable**

| Variable | Physical dysfunction | | | | | |
| --- | --- | --- | --- | --- | --- | --- |
|  | 3-month | | 6-month | | 12-month | |
|  | Odds Ratio | P value | Odds Ratio | P value | Odds Ratio | P value |
| Incidence of physical dysfunction at the previous follow-up | **13.75 [5.65-39.22]** | **<0.01** | **29.67 [11.31-88.78]** | **<0.01** | **28.92 [8.70-96.11]** | **<0.01** |
| Incidence of cognitive dysfunction at the previous follow-up | 1.45 [0.65-3.25] | 0.36 | 2.24 [0.81-6.49] | 0.12 | 1.52 [0.47-4.92] | 0.49 |
| Incidence of mental disorder at the previous follow-up | 1.16 [0.55-2.43] | 0.69 | 1.56 [0.55-4.35] | 0.40 | 0.70 [0.21-2.36] | 0.56 |
| Variable | Cognitive dysfunction | | | | | |
|  | 3-month | | 6-month |  | 12-month |  |
|  | Odds Ratio | P value | Odds Ratio | P value | Odds Ratio | P value |
| Incidence of physical dysfunction at the previous follow-up | 1.71 [0.85-3.43] | 0.13 | **3.60 [1.46-8.86]** | **0.01** | 1.11 [0.42-2.91] | 0.84 |
| Incidence of cognitive dysfunction at the previous follow-up | **3.23 [1.61-6.61]** | **0.01** | **9.04 [3.85-21.23]** | **<0.01** | **6.22 [2.67-14.48]** | **<0.01** |
| Incidence of mental disorder at the previous follow-up | 1.24 [0.63-2.43] | 0.53 | 1.84 [0.79-4.28] | 0.16 | 0.99 [0.43-2.27] | 0.98 |
| Variable | Mental disorders | | | | | |
|  | 3-month | | 6-month | | 12-month | |
|  | Odds Ratio | P value | Odds Ratio | P value | Odds Ratio | P value |
| Incidence of physical dysfunction at the previous follow-up | 1.59 [0.78-3.27] | 0.20 | 1.48 [0.66-3.37] | 0.345 | 1.34 [0.48-3.72] | 0.57 |
| Incidence of cognitive dysfunction at the previous follow-up | 1.20 [0.58-2.46] | 0.62 | 1.06 [0.49-2.27] | 0.88 | **2.71 [1.10-6.70]** | **0.03** |
| Incidence of mental disorder at the previous follow-up | **3.58 [1.81-7.35]** | **<0.01** | **6.56 [3.03-14.18]** | **<0.01** | **6.91 [2.88-16.59]** | **<0.01** |

Data in the table are presented as Odds Ratio with 95% Confidence Interval.

The variables used in the multivariable analysis were limited to the incidence of physical and cognitive dysfunction and mental disorders at the last follow-up.

**Supplemental Table 4. Association of PICS at a follow-up with its prior follow-up in the multivariable analysis with a few variables**

| Variable | Physical dysfunction | | | | | |
| --- | --- | --- | --- | --- | --- | --- |
|  | 3-month | | 6-month | | 12-month | |
|  | Odds Ratio | P value | Odds Ratio | P value | Odds Ratio | P value |
| Age | **1.04 [1.00-1.07]** | **0.04** | **1.07 [1.01-1.13]** | **0.02** | 1.07 [0.99-1.15] | 0.05 |
| Sex (Male) | 1.12 [0.51-2.45] | 0.79 | 1.27 [0.43-3.72] | 0.66 | 2.15 [0.60-7.66] | 0.24 |
| Body Mass Index | 0.97 [0.89-1.06] | 0.49 | 0.98 [0.86-1.13] | 0.80 | 1.00 [1.00-1.00] | 0.95 |
| Clinical Frailty Scale prior to hospital admission | **1.39 [1.11-1.75]** | **0.01** | 1.21 [0.88-1.68] | 0.25 | **1.74 [1.22-2.48]** | **0.02** |
| Sequential Organ Failure Assessment (SOFA) sum score | 1.09 [0.98-1.21] | 0.12 | 1.06 [0.92-1.21] | 0.41 | 0.96 [0.81-1.13] | 0.61 |
| Incidence of physical dysfunction at the previous follow-up | **8.58 [3.09-23.87]** | **<0.01** | **19.32 [6.57-56.84]** | **<0.01** | **20.38 [4.94-80.04]** | **<0.01** |
| Incidence of cognitive dysfunction at the previous follow-up | 1.08 [0.44-2.67] | 0.87 | 1.36 [0.44-4.23] | 0.60 | 0.83 [0.18-3.78] | 0.81 |
| Incidence of mental disorder at the previous follow-up | 1.36 [0.61-3.02] | 0.45 | 2.11 [0.68-6.60] | 0.20 | 0.75 [0.19-2.85] | 0.67 |
| Variable | Cognitive dysfunction | | | | | |
|  | 3-month | | 6-month |  | 12-month |  |
|  | Odds Ratio | P value | Odds Ratio | P value | Odds Ratio | P value |
| Age | **1.04 [1.01-1.07]** | **0.02** | **1.06 [1.01-1.11]** | **0.01** | **1.09 [1.03-1.14]** | **<0.01** |
| Sex (Male) | 0.73 [0.36-1.49] | 0.39 | 0.64 [0.24-1.69] | 0.37 | 1.16 [0.55-2.43] | 0.76 |
| Body Mass Index | 0.96 [0.89-1.03] | 0.26 | 1.02 [0.92-1.12] | 0.76 | 1.00 [1.00-1.00] | 0.97 |
| Clinical Frailty Scale prior to hospital admission | 1.00 [0.81-1.22] | 0.97 | 1.05 [0.79-1.40] | 0.73 | 1.00 [0.77-1.30] | 0.98 |
| Sequential Organ Failure Assessment (SOFA) sum score | 1.07 [0.98-1.17] | 0.11 | 0.94 [0.83-1.06] | 0.30 | 1.02 [0.92-1.14] | 0.65 |
| Incidence of physical dysfunction at the previous follow-up | 1.49 [0.67-3.29] | 0.32 | **2.93 [1.03-8.33]** | **0.04** | 0.64 [0.20-2.02] | 0.44 |
| Incidence of cognitive dysfunction at the previous follow-up | **2.77 [1.32-5.80]** | **0.01** | **8.83 [3.31-23.56]** | **<0.01** | **4.13 [1.61-10.57]** | **0.03** |
| Incidence of mental disorder at the previous follow-up | 1.33 [0.66-2.68] | 0.42 | 2.49 [0.96-6.46] | 0.06 | 1.23 [0.49-3.09] | 0.66 |
| Variable | Mental disorders | | | | | |
|  | 3-month | | 6-month | | 12-month | |
|  | Odds Ratio | P value | Odds Ratio | P value | Odds Ratio | P value |
| Age | **0.96 [0.93-0.99]** | **0.01** | 1.01 [0.98-1.05] | 0.52 | 0.98 [0.94-1.02] | 0.29 |
| Sex (Male) | 1.00 [0.49-2.07] | 0.99 | 1.51 [0.67-3.42] | 0.32 | 0.70 [0.26-1.86] | 0.48 |
| Body Mass Index | 1.00 [0.99-1.00] | 0.50 | 1.00 [1.00-1.00] | 0.94 | 0.97 [0.89-1.06] | 0.54 |
| Clinical Frailty Scale prior to hospital admission | **1.23 [1.00-1.51]** | **0.05** | 0.86 [0.67-1.10] | 0.22 | **1.35 [1.03-1.76]** | **0.03** |
| Sequential Organ Failure Assessment (SOFA) sum score | 1.05 [0.96-1.14] | 0.29 | 0.97 [0.88-1.07] | 0.60 | 0.95 [0.85-1.07] | 0.41 |
| Incidence of physical dysfunction at the previous follow-up | 1.61 [0.72-3.61] | 0.25 | 1.50 [0.60-3.70] | 0.39 | 1.07 [0.34-3.34] | 0.91 |
| Incidence of cognitive dysfunction at the previous follow-up | 1.44 [0.66-3.11] | 0.36 | 0.99 [0.43-2.26] | 0.99 | **3.28 [1.16-9.27]** | **0.03** |
| Incidence of mental disorder at the previous follow-up | **4.31 [2.01-9.20]** | **<0.01** | **7.75 [3.35-17.96]** | **<0.01** | **8.78 [3.30-23.36]** | **<0.01** |

Data in the table are presented as Odds Ratio with 95% Confidence Interval.

The variables used in the multivariable analysis were Age, Sex, Body Mass Index, Clinical Frailty Scale, Sequential Organ Failure Assessment (SOFA) sum score, and the incidence of physical and cognitive dysfunction and mental disorders at the last follow-up.

**Supplemental Table 5. Risk factors for physical dysfunction at each follow-up assessment.**

|  | Physical dysfunction | | | | | |
| --- | --- | --- | --- | --- | --- | --- |
|  | 3-month | | 6-month | | 12-month | |
| Variable | Odds Ratio | P value | Odds Ratio | P value | Odds Ratio | P value |
| Age (year) | 1.04 [0.99-1.10] | 0.09 | **1.13 [1.03-1.24]** | **0.01** | **1.17 [1.05-1.34]** | **0.01** |
| Sex (Male) | 0.85 [0.34-2.10] | 0.72 | 0.97 [0.27-3.43] | 0.96 | 2.86 [0.54-15.00] | 0.22 |
| Body Mass Index (kg/m^2^) | 0.97 [0.86-1.08] | 0.52 | 1.00 [0.99-1.00] | 0.96 | 1.00 [0.89-1.00] | 0.93 |
| Charlson Comorbidity Index | 1.06 [0.85-1.33] | 0.61 | 1.16 [0.82-1.65] | 0.40 | 0.98 [0.61-1.47] | 0.93 |
| Clinical Frailty Scale prior to hospital admission | 1.17 [0.89-1.55] | 0.26 | 1.04 [0.68-1.60] | 0.86 | **1.84 [1.15-3.27]** | **0.02** |
| Barthel Index prior to hospital admission | 0.97 [0.93-1.00] | 0.11 | 0.98 [0.94-1.01] | 0.21 | 1.02 [0.98-1.06] | 0.32 |
| Employment status prior to hospital admission | 0.39 [0.12-1.29] | 0.12 | 0.37 [0.07-1.9] | 0.24 | 1.37 [0.14-13.51] | 0.79 |
| ICU admission Route: |  |  |  |  |  |  |
| ER | 0.88 [0.29-2.67] | 0.82 | 1.16 [0.21-6.32] | 0.87 | 3.41 [0.36-32.77] | 0.29 |
| Source of Infection |  |  |  |  |  |  |
| Abdomen | 1.38 [0.31-6.13] | 0.67 | 1.69 [0.22-12.84] | 0.61 | 0.96 [0.03-29.01] | 0.98 |
| Respiratory | 0.100 [0.17-5.93] | 1.00 | 1.60 [0.10-25.65] | 0.74 | 0.57 [0.01-24.40] | 0.77 |
| Urinary tract | 3.54 [0.68-18.37] | 0.13 | 0.74 [0.08-6.59] | 0.78 | 0.88 [0.03-29.13] | 0.94 |
| Musculoskeletal and soft tissue | 2.03 [0.40-10.38] | 0.39 | 1.18 [0.16-8.59] | 0.87 | 2.81 [0.09-92.17] | 0.56 |
| Sequential Organ Failure Assessment (SOFA) sum score | 1.08 [0.94-1.26] | 0.29 | 1.18 [0.96-1.44] | 0.12 | 1.05 [0.80-1.37] | 0.74 |
| Septic Shock | 2.83 [0.89-9.05] | 0.08 | 0.37 [0.06-2.32] | 0.29 | 0.42 [0.05-3.58] | 0.43 |
| Lactate level at the time of ICU admission (mmol/L) | 0.98 [0.92-1.03] | 0.52 | 0.87 [0.74-1.04] | 0.13 | 0.90 [0.63-1.12] | 0.51 |
| Noninvasive positive pressure ventilation (NPPV) | 1.13 [0.27-4.70] | 0.87 | 1.46 [0.12-17.83] | 0.77 | 0.08 [0.01-1.95] | 0.12 |
| High flow nasal canula (HFNC) | 0.88 [0.26-3.00] | 0.84 | 0.41 [0.05-3.25] | 0.40 | 0.18 [0.02-2.04] | 0.17 |
| Invasive mechanical ventialtion | 0.49 [0.17-1.41] | 0.19 | 0.43 [0.10-1.87] | 0.26 | 1.26 [0.15-10.35] | 0.83 |
| Renal replacement therapy | 1.39 [0.49-3.89] | 0.54 | 1.17 [0.23-5.84] | 0.85 | 2.25 [0.26-18.99] | 0.47 |
| Length of Hospital stay (days) | **1.02 [1.00-1.03]** | **0.05** | 1.02 [0.99-1.04] | 0.06 | 1.02 [0.99-1.06] | 0.17 |
| Incidence of physical dysfunction at the previous follow-up | **7.21 [2.21-23.53]** | **<0.01** | **24.24 [6.11-96.20]** | **<0.01** | **21.41 [3.67-124.85]** | **<0.01** |
| Incidence of cognitive dysfunction at the previous follow-up | 1.22 [0.41-3.65] | 0.72 | 0.78 [0.20-3.04] | 0.73 | 1.22 [0.16-9.07] | 0.85 |
| Incidence of mental disorder at the previous follow-up | 1.42 [0.57-3.54] | 0.46 | 3.15 [0.75-13.26] | 0.12 | 1.12 [0.19-6.55] | 0.90 |

ER: emergency room, ICU: Intensive Care Unit

**Supplemental Table 6. Risk factors for cognitive dysfunction at each follow-up**

|  | Cognitive dysfunction | | | | | |
| --- | --- | --- | --- | --- | --- | --- |
|  | 3-month | | 6-month | | 12-month | |
| Variable | Odds Ratio | P value | Odds Ratio | P value | Odds Ratio | P value |
| Age (year) | **1.05 [1.00-1.93]** | **0.02** | **1.08 [1.01-1.16]** | **0.03** | **1.08 [1.02-1.15]** | **0.01** |
| Sex (Male) | 0.59 [0.25-1.39] | 0.23 | 0.65 [0.20-2.17] | 0.49 | 0.64 [0.21-1.90] | 0.42 |
| Body Mass Index (kg/m^2^) | 0.99 [0.90-1.08] | 0.77 | 1.07 [0.95-1.20] | 0.28 | 1.03 [0.93-1.15] | 0.57 |
| Charlson Comorbidity Index | 1.17 [0.93-1.50] | 0.20 | 1.22 [0.84-1.83] | 0.33 | 1.14 [0.83-1.60] | 0.44 |
| Clinical Frailty Scale prior to hospital admission | 0.92 [0.70-1.21] | 0.57 | 1.37 [0.90-2.09] | 0.15 | 0.92 [0.63-1.29] | 0.62 |
| Barthel Index prior to hospital admission | **0.94 [0.90-0.98]** | **0.01** | 1.02 [0.98-1.06] | 0.33 | 1.00 [0.96-1.03] | 0.78 |
| Employment status prior to hospital admission | 0.61 [0.23-1.64] | 0.33 | 2.19 [0.57-8.34] | 0.25 | 0.31 [0.09-1.00] | 0.05 |
| ICU admission Route: |  |  |  |  |  |  |
| ER | 1.63 [0.62-4.29] | 0.33 | 0.86 [0.17-4.23] | 0.85 | 1.05 [0.29-3.81] | 0.94 |
| Source of Infection |  |  |  |  |  |  |
| Abdomen | 0.60 [0.18-2.04] | 0.41 | 0.50 [0.09-2.78] | 0.43 | 2.09 [0.44-9.88] | 0.36 |
| Respiratory | 0.43 [0.10-1.85] | 0.26 | 1.06 [0.16-7.15] | 0.95 | 1.92 [0.32-11.41] | 0.47 |
| Urinary tract | 0.54 [0.12-2.50] | 0.43 | **0.07 [0.01-0.76]** | **0.03** | 1.67 [0.25-11.39] | 0.60 |
| Musculoskeletal and soft tissue | **0.19 [0.04-0.83]** | **0.03** | 0.22 [0.03-1.78] | 0.16 | 1.70 [0.27-10.49] | 0.57 |
| Sequential Organ Failure Assessment (SOFA) sum score | 1.07 [0.95-1.22] | 0.28 | 0.94 [0.78-1.12] | 0.48 | 1.11 [0.96-1.30] | 0.16 |
| Septic Shock | 0.78 [0.27-2.28] | 0.66 | 3.10 [0.67-14.38] | 0.15 | **0.24 [0.07-0.86]** | **0.03** |
| Lactate level at the time of ICU admission (mmol/L) | 1.02 [0.98-1.06] | 0.31 | 0.99 [0.94-1.04] | 0.71 | 0.99 [0.94-1.05] | 0.78 |
| Noninvasive positive pressure ventilation (NPPV) | 0.28 [0.07-1.12] | 0.07 | **6.63 [1.17-37.46]** | **0.03** | 0.58 [0.11-3.06] | 0.52 |
| High flow nasal canula (HFNC) | 1.63 [0.54-4.91] | 0.38 | 0.69 [0.15-3.08] | 0.63 | 0.91 [0.24-3.35] | 0.88 |
| Invasive mechanical ventialtion | 0.81 [0.31-2.15] | 0.67 | 0.32 [0.07-1.56] | 0.16 | 0.85 [0.24-3.03] | 0.80 |
| Renal replacement therapy | 1.34 [0.54-3.30] | 0.53 | **0.20 [0.04-0.88]** | **0.03** | 0.52 [0.15-1.78] | 0.29 |
| Length of Hospital stay (days) | 1.00 [0.98-1.01] | 0.39 | 1.00 [0.98-1.01] | 0.85 | 1.01 [0.99-1.02] | 0.24 |
| Incidence of physical dysfunction at the previous follow-up | 1.31 [0.53-3.23] | 0.56 | **5.19 [1.31-20.61]** | **0.02** | 0.41 [0.10-1.65] | 0.21 |
| Incidence of cognitive dysfunction at the previous follow-up | **3.97 [1.59-9.86]** | **<0.01** | **16.29 [4.39-60.41]** | **<0.01** | **4.12 [1.37-12.41]** | **0.01** |
| Incidence of mental disorder at the previous follow-up | 1.08 [0.48-2.41] | 0.86 | **3.83 [1.05-14.02]** | **0.04** | 1.11 [0.37-3.30] | 0.86 |

ER: emergency room, ICU: Intensive Care Unit

**Supplemental Table 7. Risk factors for mental disorders at each follow-up**

|  | Mental disorder | | | | | |
| --- | --- | --- | --- | --- | --- | --- |
|  | 3-month | | 6-month | | 12-month | |
| Variable | Odds Ratio | P value | Odds Ratio | P value | Odds Ratio | P value |
| Age (year) | **0.93 [0.88-0.97]** | **<0.01** | 1.01 [0.97-1.07] | 0.59 | 0.95 [0.90-1.00] | 0.06 |
| Sex (Male) | 0.79 [0.34-1.82] | 0.58 | 1.11 [0.40-3.06] | 0.84 | 0.35 [0.10-1.23] | 0.10 |
| Body Mass Index (kg/m^2^) | 1.00 [0.96-1.00] | 0.95 | 1.00 [0.95-1.00] | 0.96 | 0.96 [0.86-1.07] | 0.44 |
| Charlson Comorbidity Index | 1.25 [1.00-1.58] | 0.05 | 1.04 [0.78-1.40] | 0.81 | 0.88 [0.63-1.23] | 0.45 |
| Clinical Frailty Scale prior to hospital admission | 1.18 [0.91-1.55] | 0.22 | **0.62 [0.43-0.87]** | **0.01** | 1.23 [0.82-1.82] | 0.32 |
| Barthel Index prior to hospital admission | 1.00 [0.97-1.03] | 0.97 | **0.96 [0.92-0.99]** | **0.03** | 0.98 [0.94-1.01] | 0.25 |
| Employment status prior to hospital admission | **0.31 [0.11-0.89]** | **0.03** | 1.36 [0.47-3.95] | 0.57 | 0.43 [0.11-1.70] | 0.23 |
| ICU admission Route: |  |  |  |  |  |  |
| ER | 1.98 [0.71-5.52] | 0.19 | 3.43 [0.95-12.36] | 0.06 | **5.67 [1.03-31.18]** | **0.05** |
| Source of Infection |  |  |  |  |  |  |
| Abdomen | 3.91 [0.99-15.35] | 0.05 | 0.55 [0.13-2.32] | 0.42 | 2.46 [0.47-12.87] | 0.29 |
| Respiratory | 2.18 [0.45-10.45] | 0.33 | 1.53 [0.29-8.16] | 0.62 | 0.39 [0.05-3.01] | 0.37 |
| Urinary tract | 2.44 [0.49-12.19] | 0.28 | 1.25 [0.20-7.72] | 0.81 | 1.10 [0.13-9.31] | 0.93 |
| Musculoskeletal and soft tissue | 3.08 [0.72-13.05] | 0.13 | 0.39 [0.08-2.07] | 0.27 | 0.66 [0.09-4.74] | 0.68 |
| Sequential Organ Failure Assessment (SOFA) sum score | **1.16 [1.01-1.34]** | **0.04** | 1.00 [0.87-1.14] | 0.99 | 0.92 [0.78-1.10] | 0.37 |
| Septic Shock | 0.63 [0.21-1.86] | 0.40 | **0.18 [0.04-0.70]** | **0.01** | 0.43 [0.09-1.95] | 0.27 |
| Lactate level at the time of ICU admission (mmol/L) | 1.00 [ 0.95-1.04] | 0.83 | 1.00 [0.95-1.05] | 0.96 | 1.01 [0.95-1.07] | 0.76 |
| Noninvasive positive pressure ventilation (NPPV) | 0.83 [0.21-3.25] | 0.79 | 2.93 [0.66-12.94] | 0.16 | 3.35 [0.57-19.78] | 0.18 |
| High flow nasal canula (HFNC) | **3.68 [1.18-11.49]** | **0.03** | 1.99 [0.58-6.89] | 0.28 | **6.35 [1.32-30.63]** | **0.02** |
| Invasive mechanical ventialtion | **0.26 [0.09-0.74]** | **0.01** | 3.50 [0.91-13.46] | 0.07 | 0.79 [0.18-3.53] | 0.76 |
| Renal replacement therapy | 0.37 [0.13-1.04] | 0.06 | 0.40 [0.13-1.22] | 0.11 | 0.50 [0.11-2.24] | 0.36 |
| Length of Hospital stay (days) | 1.01 [0.99-1.02] | 0.23 | 1.01 [0.99-1.02] | 0.32 | 1.01 [0.99-1.02] | 0.56 |
| Incidence of physical dysfunction at the previous follow-up | 2.29 [0.84-6.21] | 0.11 | 2.09 [0.68-6.40] | 0.20 | 0.95 [0.23-3.95] | 0.95 |
| Incidence of cognitive dysfunction at the previous follow-up | 1.77 [0.69-4.55] | 0.24 | 0.69 [0.25-1.88] | 0.46 | **4.23 [1.24-14.44]** | **0.02** |
| Incidence of mental disorder at the previous follow-up | **4.30 [1.77-10.42]** | **<0.01** | **10.37 [3.59-29.97]** | **<0.01** | **7.18 [2.21-23.34]** | **<0.01** |

ER: emergency room, ICU: Intensive Care Unit

**Supplemental Table 8. Differences in patient characteristics from past papers**

| Country | Japan | ANZICS | Europe (Germn) | US | Netherland |
| --- | --- | --- | --- | --- | --- |
| n | 339 | 282 | 753 | 60 | 246 |
| Age | 74 [67-82] | 60.1 (47.0–69.8) | 65 [56-74] | 59±13 (24–88) | 61.2 (9.3) |
| Body Mass Index | 23.4 [20.4-25.8] | 27.0 (23.5–31.2) | - | 31.2±5.2 | 28.0 (4.5) |
| Reference | (1) | (2) | (3) | (4) | (5) |

Reference (1): Liu K, Nakashima K, Goto T, Nakamura K, Nakano H, Motoki M, et al. Investigating Long term Outcomes of ICU patients with Sepsis or Septic shock (ILOSS) Study Group: Phenotypes of functional decline or recovery in sepsis ICU survivors: Insights from a 1-year follow-up multicenter cohort analysis. Crit Care Med. 2025; doi: 10.1097/CCM.0000000000006621 (Ahead of print)

Reference (2): Hodgson CL, Higgins AM, Bailey M, Barrett J, Bellomo R, Cooper DJ, et al. Comparison of 6-month outcomes of sepsis versus non-sepsis critically ill patients receiving mechanical ventilation. Crit Care. 2022;26:174.

Reference (3): Fleischmann-Struzek C, Born S, Kesselmeier M, Ely EW, Töpfer K, Romeike H, et al. Functional dependence following intensive care unit-treated sepsis: three-year follow-up results from the prospective Mid-German Sepsis Cohort (MSC). Lancet Reg Health Eur. 2024;46:101066.

Reference (4): Maley JH, Sandsmark DK, Trainor A, Bass GD, Dabrowski CL, Magdamo BA, et al. Six-Month Impairment in Cognition, Mental Health, and Physical Function Following COVID-19-Associated Respiratory Failure. Crit Care Explor. 2022;4:e0673.

Reference (5): Mesina RS Jr, Rustøen T, Hagen M, Laake JH, Hofsø K. Long-term functional disabilities in intensive care unit survivors: A prospective cohort study. Aust Crit Care. Aust Crit Care. 2024;37:843-50.
